# Effect of two Different Concentrations of Propofol and Ketamine Combinations (Ketofol) in Pediatric Patients under Lumbar Puncture or Bone Marrow Aspiration

**Published:** 2013-01-22

**Authors:** A Ghadami Yazdi, V Ayatollahi, A Hashemi, SH Behdad, E Ghadami Yazdi

**Affiliations:** 1 Department of Anesthesia, Shahid Sadoughi University of Medical Sciences and Health Services, Yazd, Iran.; 2 Department of Pediatrics, Hematology, Oncology and Genetics Research Center, Shahid Sadoughi University of Medical Sciences and Health Services, Yazd, Iran.; 3 Resident of Iranian Traditional Medicine, Tehran University of Medical Sciences Tehran, Iran.

**Keywords:** Ketamine, Propofol, Spinal Puncture, Pediatrics

## Abstract

**Background:**

Ketamine is an anesthetic drug that is importantly analgesic without respiratory depression. Ketamine increases blood pressure and heart rate. Propofol is an anesthetic drug with good sedation, rapid recovery, but it causes respiratory depression, low heart rate and low blood pressure. Combination of Ketamine and Propofol provides sedation, analgesia and rapid recovery with hemodynamic stability and minimal respiratory depression. The aim of this study was to compare two different combinations of these two drugs to reach necessary sedation scale for the Lp or BMA in pediatric with ALL.

**Materials and Methods:**

This randomized, double blinded study was designed to compare the quality of sedation and side effects of intravenous Ketofol on 60 patients of both gender. Patients received titrated injection of a solution containing combination of one part of Ketamine and two parts of Propofol (1:2) (group I) or one part of Ketamine and three parts of Propofol (1:3) (group II) to reach almost near 5 sedation level (using Ramsay Sedation Scale). Respiratory and hemodynamic profiles, amount of drug injected and side effects were recorded.

**Results:**

These drug combinations were used on 60 children with a median age of 6.2 years. In this study, recovery time and hallucination was significantly high in group I, but in both groups hemodynamic were stable, amnesia was enough, and there was no respiratory depression.

**Conclusion:**

Lower doses of Ketamine in these combinations have lower psycho mimetic side effects, and shorter recovery time.

## Introduction

The lumbar puncture (LP) or bone marrow aspiration (BMA) in pediatric patients with hematological diseases is often repeated at regular intervals (1, 2). These procedures are painful and unpleasant for the children, and a good sedation is essential in these procedures (1, 2). An ideal sedation agent should not only have rapid onset and a smooth recovery period, but should also provide sufficient analgesia, sedation with adequate cardiovascular and respiratory function, amnesia, and motor control immobile throughout the procedure (3). Ketamine is an anesthetic drug that is importantly analgesic without respiratory depression. Ketamine increases blood pressure and heart rate (3, 4). Propofol is an anesthetic drug with good sedation and rapid recovery time, but also as a respiratory depression that decreases heart rate and blood pressure (3, 4). Combination of Ketamine and Propofol provides sedation, analgesia and rapid recovery with hemodynamic stability and minimal respiratory depression (3-10). In a previous study, we compared the effects of two types of Ketofol mixtures, equal amounts of Propofol and Ketamine (1:1) or two parts of Propofol plus one part of Ketamine (2:1) (4). We observed that combination of 2:1 was better than 1:1, because this combination minimizes the psycho mimetic side effects and shortens the recovery time. The aim of this study was to compare total doses, recovery time, and adverse effects of combinations of Ketamine and Propofol with different ratios (1:2 and 1:3) for necessary sedation scale in the LP or BMA in pediatrics. 

## Materials and Methods

In this randomized and double blinded study, 60 children of 3 to 12 years old with ALL who were referred to a pediatric hospital in Yazd for the BMA or LP were studied. First, the patients and their parents need to know about the type of sedation method was given. Then Informed consent was obtained. Inclusion criteria were all consecutive children with hematological diseases undergoing BMA or lumbar puncture admitted to the hospital. Exclusion criteria included: prior sensitization or allergic reaction to Propofol, Ketamine, soy or egg products; hypotension head injury, increased intracranial or intraocular pressure; use of drugs known to interact with either study agent. Patients with heart disease, cardiovascular, respiratory, hepatic, and epilepsy were excluded. Patients were randomly divided into two groups of 30. In the waiting room, IV catheter was placed with using Emla cream for the patient. Primary measures of RR, HR, mean arterial pressure, and oxygen saturation were recorded combination of Propofol - Ketamine was provided by an anesthesia nurse who was not involved in this case study. For Group I, 25 mg Ketamine with 50 mg Propofol in the 5.5 cc and for Group II, 50 mg Propofol plus 17 mg Ketamine with distilled water to volume 5.5 cc was completed. Then coded syringes were given to resident of anesthesia. Drug was injected 0.07 cc/kg slowly, and then repeated to reach of 5 Ramsay sedation scale. Oxygen saturation, mean arterial pressure, heart rate and respiratory rate were measured continuously, and results were recorded in the three different times (after injection, the time of the procedure, and the time of the patient's arrival to the recovery room). If patient had pain or movement during the procedure, drug injection was repeated and repeated doses were noted. Total drug usage was calculated at the end of trial. Procedure duration was recorded. After the procedure, patients were taken to the recovery room until they reach Aldrete score of 9 to 10. Recovery time from the end of the procedure to reach Aldrete score of 9 to 10 was considered, and noted. Nausea, vomiting and other side effects including hallucinations, agitation and respiratory depression were listed Vital signs of patients were evaluated every 5 minutes. When the patients had stable vital signs, they were alert and able to move without assistance, and some symptoms such as nausea and vomiting were not sensible, they discharged from recovery room. Patients were monitored for at least 2 hours after the procedure. All data include sex, age and weight recorded in a standard data collection record.

**Statistical Analysis**

All data were analyzed by SPSS software version 15 with Student t-test, Fisher-exact, and Mann-Whitney.

## Results

Analyzed data of 60 patients showed that demographic characteristics (number, age, sex and weight) were similar in two groups, and there were no significant differences among two groups (Table I). The mean of procedure durations were not different in both groups (P-value= 0.57), but mean of recovery time was longer in group I. This difference was significant (P-value <0.001). Mean of recovery time in group I and II was 11.30 min and 8.03 min, respectively. Three patients in group I and one patient in group II experienced nausea (P-value= 0.41). Seven patients in group I and 1 in group II had hallucination (P-value= 0.05). No respiratory problem and vomiting were seen in both groups. None of the children had bad memory, and all were satisfied with the procedure. Table II shows that Ramsay scores were similar in two groups after injection of 0.07 ml/kg, and additional drug was required to achieve Ramsay score of 5. The amount of next injection was significantly different in two groups, group II required more than group I. Total volume of injected drugs and total Ketamine were similar, but total Propofol in group II was more than group I (P-value <0.001). Mean of Spo2, HR, MAP and RR were similar for different times (Table II).

**Table I:** Demographic, preoperative and side effects data

	**Group I ** ** ( K/P 1/2)**	**Group II ** ** (K/P 1/3)**	**P- value**	**Test**
**Sex (M/F)**	23/7	21/9	0.77	Fisher-exact
**Mean Age (yr)**	6.1	6.3	0.78	T-test
**Mean Weight (kg)**	17.28	16.88	0.83	T-test
**Procedure** ** duration(min)**	4.4	4.5	0.57	T-test
**Recovery duration (min)**	11.30	8.03	<0.001	T-test
**Nausea**	3	1	0.41	Fisher exact
**Hallucination**	7	1	0.05	Fisher exact

**Table II:** Data and Results of drugs in two groups

**Mean of**	**Group I ** ** ( K/P 1/2)**	**Group II ** ** (K/P 1/3)**	**P- value**	**Test**
**Ramsay score after first injection 0.07 cc/kg**	4.43	4.17	0.113	Mann-Whitney
**The next injection amount (cc)**	0.58	1.18	<0.001	Mann-Whitney
**Total volume of injected drugs (cc)**	1.8	2.35	0.027	T-test
**Total Propofol (mg/kg)**	0.87	1.16	<0.001	T-test
**Total Ketamine (mg/kg)**	0.43	0.39	0.48	T-test

**Table III:** Patient’s vital signs (T test)

**Mean of**	**Group I ** ** ( K/P 1/2)**	**Group II ** ** (K/P 1/3)**	**P- value**
**SPO2 T1**	96.6	96.7	0.41
** T2**	96.9	96.8	0.84
** T3**	96.7	97.0	0.35
**HR T1**	116.6	114.9	0.40
** T2**	115.7	115.5	0.93
** T3**	109.4	109.4	0.123
**MAP T1**	63.0	62.0	0.56
** T2**	64.6	63.5	0.57
** T3**	62.1	57.4	0.005
**RR T1**	20.1	18.6	0.02
** T2**	20.0	19.5	0.33
** T3**	18.8	17.2	0.005

T1: After drug injection -T2: Time of the procedure- T3: Time of the patient's arrival to the recovery room

## Discussion

BMA and LP are two processes that should be repeated in children with ALL. Both process are painful and unpleasant, and can cause fear and anxiety in children, especially in later process (1, 2 and 4). The ideal medication provides analgesia, sedation, amnesia, and has not adverse effects on hemodynamic and respiratory system. Also it should have minimal additional side effects (3). There is not a drug with all of these specifications, and we have to use the multiple drugs (3). Ketamine is an anesthetic drug that produces analgesia and amnesia without respiratory depression. Ketamine increases hemodynamic parameters non-dose-dependently, but it sometimes causes nausea, vomiting or hallucination (3-13). Propofol is an anesthetic drug with good sedation, low amnesia and rapid recovery, but respiratory depression. Propofol decreases heart rate and blood pressure dose –dependently (3-13). The combination of these two drugs can cause sedation, analgesia, amnesia and rapid recovery with hemodynamic stability and minimal respiratory depression (4-13).

Our previous study (2011) was performed using a mixture of the 1:1 compared1:2Ketamine Propofol. Nausea, hallucination and recovery time were also more in1:1 ratio (4). Due to the better results of 1:2 ratio in comparison with 1:1 ratio it was more preferred to compare 1:2 with 1:3 ratio. In this study, we observed that 1:3 ratio was better than 1:2, because it had a shorter recovery time, and total drug usage was reduced in this group. Incidence of hallucination and nausea were lower, although were not statically significant. Bardineath et al reported that ratio of 1:5 Ketofol with local anesthesia provides good analgesia and sedation for breast biopsy procedure (5). Andolfatto G et al reported 1:1 ratio of Ketofol is highly effective in pediatric emergency procedures (6). The results are different. Mean recovery time in their study was 14 min but it was 11.3 min for ratio 1:2 and 8.0 min for 1:3 in our study. Then low level of Ketamine decreases recovery time with good sedation and analgesia. Silva PS et al used Ketofol of 1:1 to reach Ramsay score of 3-4in children with hematological diseases (7). The mean total dose of Ketofol administered was 1.25 mg/kg per each of Propofol and Ketamine while it was lower in our study to reach higher Ramsay score of 5. So, using of high dose Ketamine cannot help to increase sedation and analgesia. Whetheral A et al observed that Ketofol infusion successfully produced deep sedation for prolonged (153 min) pediatric orthopedic procedures in conjunction with regional analgesia (8). Similar to our study, they reported that Ketofol is safe and good combination for children. Erdogan kayhan G et al compared Ketofol1:1 with Propofol in electroconvulsive therapy. Ketofol had better results in their study (9). Erdogan Ma et al compaired Propofol with Ketofol in laryngeal mask insertion in elderly patient (10). They showed that the number of patients who required ephedrine and the total ephedrine dose were lower, and apnea duration was increased in the Ketofol group. Ephedrine treat hypotension and bradycardia due to Propofol and Ketamine can also do so. But increased apnea duration in their study could be related to high age. So, Ketamine similar to ephedrine improves the hemodynamic effects of Propofol. Phillips w et al studied the effect of Propofol versus Propofol/Ketamine for brief painful procedures (11). Similar to our study, none of the patient in either group had respiratory depression or required any intervention. The combination of Propofol and Ketamine provided an appropriate combination for painful procedural sedation in the emergency department. Compared to Propofol alone, Ketofol provided less hypotension, better sedation, and increased patient comfort and safety. In another study by Andolfatto G et al, sedation and analgesia effects of Propofol alone versus Ketofol were compared (12). Ketofol did not show any result regarding to reduce incidence of adverse respiratory events compared with Propofol alone. Induction time, efficacy and sedation time were similar but, sedation depth presented to be more consistent with Ketofol. In this study we showed, the combination of the two drugs reduced the dose of each drug alone. Smischney NG et al studied hemodynamic effects of Ketofol in induction of general anesthesia (13). They observed that Ketofol improved hemodynamic during the first 10 minutes after induction, and it was a good induction agent. This finding is consistent with hemodynamic stability in our study.

## Conclusion

According to this finding, Ketofol is a good combination for sedation and analgesia in painful short procedures in children. This mixture has hemodynamic and respiratory safety. Use of low Ketamine in this combination decreases recovery time with minimal side effects such as nausea and hallucination. Combination of 1:3 Ketamine-Propofol is an appropriate ratio, and we mentioned the use of it.
